# Patient-provider discordance between global assessments of disease activity in rheumatoid arthritis: a comprehensive clinical evaluation

**DOI:** 10.1186/s13075-017-1419-5

**Published:** 2017-09-26

**Authors:** Divya N. Challa, Zoran Kvrgic, Andrea L. Cheville, Cynthia S. Crowson, Tim Bongartz, Thomas G. Mason, Eric L. Matteson, Clement J. Michet, Scott T. Persellin, Daniel E. Schaffer, Theresa L. Wampler Muskardin, Kerry Wright, John M. Davis

**Affiliations:** 1 Division of Rheumatology, Mayo Clinic, 200 First St. SW, Rochester, MN 55905 USA; 2 Department of Physical Medicine and Rehabilitation, Mayo Clinic, 200 First St. SW, Rochester, MN 55905 USA; 3 Division of Biostatistics and Informatics, Mayo Clinic, 200 First St. SW, Rochester, MN 55905 USA; 4 Department of Emergency Medicine, Vanderbilt University Medical Center, Nashville, TN USA

**Keywords:** Depression, Disease activity, Fibromyalgia, Patient-reported outcomes, Rheumatoid arthritis

## Abstract

**Background:**

Discordance between patients with rheumatoid arthritis (RA) and their rheumatology health care providers is a common and important problem. The objective of this study was to perform a comprehensive clinical evaluation of patient-provider discordance in RA.

**Methods:**

A cross-sectional observational study was conducted of consecutive RA patients in a regional practice with an absolute difference of ≥ 25 points between patient and provider global assessments (possible points, 0–100). Data were collected for disease activity measures, clinical characteristics, comorbidities, and medications. In a prospective substudy, participants completed patient-reported outcome measures and underwent ultrasonographic assessment of synovial inflammation. Differences between the discordant and concordant groups were tested using χ^2^ and rank sum tests. Multivariable logistic regression was used to develop a clinical model of discordance.

**Results:**

Patient-provider discordance affected 114 (32.5%) of 350 consecutive patients. Of the total population, 103 patients (29.5%) rated disease activity higher than their providers (i.e., ‘positive’ discordance); only 11 (3.1%) rated disease activity lower than their providers and were excluded from further analysis. Positive discordance correlated with negative rheumatoid factor and anticyclic citrullinated peptide antibodies, lack of joint erosions, presence of comorbid fibromyalgia or depression, and use of opioids, antidepressants, or anxiolytics, or fibromyalgia medications. In the prospective study, the group with positive discordance was distinguished by higher pain intensity, neuropathic type pain, chronic widespread pain and associated polysymptomatic distress, and limited functional health status. Depression was found to be an important mediator of positive discordance in low disease activity whereas the widespread pain index was an important mediator of positive discordance in moderate-to-high disease activity states. Ultrasonography scores did not reveal significant differences in synovial inflammation between discordant and concordant groups.

**Conclusions:**

The findings provide a deeper understanding of patient-provider discordance than previously known. New insights from this study include the evidence that positive discordance is not associated with unrecognized joint inflammation by ultrasonography and that depression and fibromyalgia appear to play distinct roles in determining positive discordance. Further work is necessary to develop a comprehensive framework for patient-centered evaluation and management of RA and associated comorbidities in patients in the scenario of patient-provider discordance.

## Background

Discordance between patients with rheumatoid arthritis (RA) and rheumatology health care providers in assessment of disease activity is an important clinical problem. By comparing the patient and provider global assessments of disease activity on visual or numerical rating scales, patient-provider discordance occurs in approximately 40% of clinical visits [1]. Most commonly, the patient global assessment is substantially higher than the provider global assessment, which is defined as ‘positive discordance’ [1–3]. Its associations with impairment in physical function and health-related quality of life highlight the clinical importance of positive discordance [1, 3, 4]. Although more prospective longitudinal studies are needed, current evidence suggests that discordance contributes to decreased work productivity, decreased likelihood of remission, and potentially increased risk of radiographic joint damage [4–6].

Many studies have sought to identify the determinants of patient-provider discordance in RA (see [1] for a systematic review). Previous cross-sectional studies have reported that patient-reported measures of pain intensity, fatigue, depression, physical function, and health-related quality of life are key correlates of discordance [4, 5]. One study has shown that discordance-associated comorbidities include aging, fibromyalgia, and osteoarthritis [5]. Whereas the patient global assessment mainly reflects pain, fatigue, and the impact of disease on overall health, the provider global assessment primarily denotes objective disease assessments, particularly swollen joints and inflammatory markers [2, 7].

However, few studies have performed a comprehensive assessment of the many clinical domains of patient-provider discordance in a way that facilitates clinical management decision making. First, previous studies have evaluated only pain intensity without consideration for underlying mechanisms or associated comorbidities. Second, there is an unmet clinical need to evaluate patient-reported outcome measures for discordance domains that are clinically actionable by informing specific management decisions. Third, no studies have yet evaluated disease activity in patients with positive discordance using a highly sensitive measure of synovial inflammation (i.e., ultrasonography). Therefore, this study aimed to delve deeper into the clinical correlates of patient-provider discordance by explicating the potential etiologic domains underlying the adverse health status of patients.

## Methods

### Study design and populations

A cross-sectional, observational study was conducted at the Mayo Clinic in Rochester, Minnesota, USA. The study population consisted of 350 patients with a diagnosis of RA or inflammatory polyarthritis by a rheumatologist. The research study coordinator (ZK) retrospectively identified patients who were seen for an appointment in the outpatient rheumatology practice within 4 weeks of screening. The coordinator abstracted data from the historical electronic medical records to ascertain the American College of Rheumatology/European League Against Rheumatism (ACR/EULAR) 2010 classification criteria for RA [8]. Eligibility required age ≥ 18 years, fulfillment of the ACR/EULAR 2010 criteria, residence within 150 miles of the clinic, and ≥ 2 outpatient follow-up appointments in the prior 18 months. Eligible patients were enrolled consecutively between 29 September 2014 and 21 May 2015.

At each clinical appointment in the Division of Rheumatology, patients completed a global assessment of disease activity on a visual analog scale (VAS) of 0 to 100 by answering the question “Considering all of the ways your disease affects you, how well are you doing in the past week?” The anchors of the VAS are “Best possible” and “Worst possible.” Providers complete their global assessment in the electronic health record on the VAS with demarcations at every 5 points. On the basis of data from the most recent visit, patient-provider discordance was defined as an absolute difference of ≥ 25 points between the patient and provider global assessments. A priori analyses determined the proportions of visits for which either the patient or provider global assessment was comparatively higher.

In addition, a prospective cross-sectional substudy was performed. The aims of this were to both identify clinical tools for assessing patients with discordance beyond standard clinical evaluation and to identify new mechanistic determinants. The study coordinator approached consecutive patients using a telephone script and invited them to participate in the study. Enrollment was stratified into three groups; 50 were required to have patient-provider discordance; 10 have patient-provider concordance and low disease activity (LDA); and 10 have patient-provider concordance and moderate-to-high disease activity (MHDA). There were no additional selection criteria.

### Data collection

Two investigators abstracted the electronic health records of patients using a standardized case report form. Data from the most recent clinical rheumatology visit included demographic characteristics, provider type (i.e., physician, fellow, nurse practitioner, or physician assistant), patient and provider global VAS scores, tender and swollen joint counts of 0 to 28, use of synthetic or biologic disease-modifying antirheumatic drugs (DMARDs), and use of anxiolytics or antidepressants, fibromyalgia medications, opioid pain medications, or sleep aids. All prior records were reviewed and data were collected for joint erosions on plain radiographs of the hands and feet, serologic test results for rheumatoid factor (RF) and anticyclic citrullinated peptide (anti-CCP) antibodies, and associated physician-diagnosed comorbidities, including anxiety and depression, fibromyalgia, obstructive sleep apnea, and osteoarthritis.

### Disease activity assessment

Disease activity was measured with the Disease Activity Score 28 using C-reactive protein (DAS28-CRP) [9, 10] and was also classified by the Clinical Disease Activity Index (CDAI) into the following groups: remission (<2.8), LDA (≥ 2.8 to < 10.0), moderate disease activity (MDA) (≥ 10.0 to < 22.0), and high disease activity (HDA) (≥ 22.0) [11].

### Patient-reported outcomes

Participants in the prospective substudy completed questionnaires at the research study visit. The Short-Form McGill Pain Questionnaire 2 (SF-MPQ-2) assesses pain descriptors, including six continuous (e.g., aching, throbbing), six intermittent (e.g., stabbing, piercing), six neuropathic (e.g., hot, burning), and four affective (e.g., punishing, fearful) descriptors, rated on an intensity scale of 0 to 3 [12]. The Fibromyalgia Research Survey includes the Widespread Pain Index (WPI), a measure of the number of painful body regions, and the Symptom Severity (SS) score, a measure of fatigue, unrefreshing sleep, cognitive symptoms, and other somatic symptoms [13]. The Polysymptomatic Distress Scale is the sum of WPI and SS scores on a continuous scale [14]. The Bristol Rheumatoid Arthritis Fatigue score assesses numeric rating scales from 0 to 10 for fatigue severity, effect, and coping, as well as a total score [15].

The Patient Health Questionnaire 9 (PHQ-9) is a reliable and valid measure of depression severity that scores each of the nine Diagnostic and Statistical Manual of Mental Disorders (Fourth Edition) criteria from 0 (not at all) to 3 (nearly every day) [16]. The protocol approved by the Mayo Clinic Institutional Review Board required that patients with PHQ-9 scores ≥ 10 be evaluated by the investigator and be given the option of primary care provider or psychiatry referral. The Generalized Anxiety Disorder 7 is a seven-item scale used to measure generalized anxiety symptoms [17]. The Mindfulness Attention and Awareness Scale is a 15-item scale designed to assess core characteristics of dispositional mindfulness and attention that are predictive of self-regulation and well-being [18]. Assessments were made from the Patient-Reported Outcomes Measurement Information System (PROMIS) 8a short forms for pain interference (version 1.0), sleep disturbance (version 1.0), fatigue (version 1.0), and ability to participate in social roles and activities (version 2.0). Each PROMIS instrument has eight items, rated by patients as 1 through 5, and total scores are obtained for each of the four instruments separately. The Health Assessment Questionnaire II (HAQ-II) is a widely used, reliable, and valid 10-item questionnaire that measures functional status [19].

### Ultrasonography

Certified rheumatologist sonographers (TB, KW, and JMD), to whom the clinical status of patients was masked, performed ultrasonographic examination of the clinically dominant hand and foot, including second and third proximal interphalangeal joints, second and third metacarpophalangeal joints, wrist, and second and fifth metatarsophalangeal joints, according to the German Ultrasound 7 score [20–22]. This method scores gray scale (GS) and power Doppler (PD) synovitis semiquantitatively (score, 0–3), as well as the presence of GS and PD tenosynovitis and erosions. Its scoring ranges are 0 to 27 for GS synovitis, 0 to 39 for PD synovitis, 0 to 7 for GS tenosynovitis, 0 to 21 for PD tenosynovitis, and 0 to 14 for erosions. These examinations were generally performed on the same day as the questionnaires.

### Statistical methods

Descriptive statistics (e.g., median, percentage) were used to summarize the data. Comparisons between the total discordant group and total concordant group and stratifications with LDA versus MHDA categories were performed using χ^2^ and rank sum tests. Multivariable logistic regression models of discordance compared with concordance were also used. Expert consensus was used to select a priori the list of variables to include in the multivariable model. Analyses were performed with SAS software version 9.4 (SAS Institute Inc.).

## Results

**Fig. 1:**
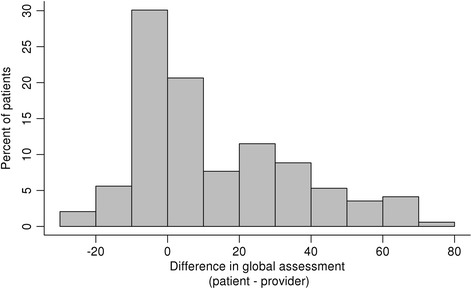
Distribution of the continuous differences between the patient and provider global assessments of disease activity in the overall study population

The study cohort included 350 consecutive patients (mean age, 63.5 years; female, 70%) with mean disease duration of 7.7 years (Table 1). Patient-provider discordance, defined by an absolute difference of ≥ 25 points between the patient and provider global assessments (Fig. 1), occurred in 114 (32.5%) of the patients, and 103 patients (29.4%) rated their global assessments higher than their providers. The 11 patients who recorded their global assessments lower than their providers were excluded from further analysis. Thus, the study focused on the 103 patients in the discordant group and 236 patients in the concordant group.

**Table 1:** Association of patient characteristics with patient-provider discordance in global assessments of disease activity in the total study population

Characteristic	All patients (*N* = 339)	Concordant group (*n* = 236)	Discordant group^a^ (*n* = 103)	*P* value
Age, years	63.5 (55.1–72.8)	62.4 (55.1–73.8)	63.5 (54.9–72.0)	.79
Sex				.15
Female	235 (69)	158 (67)	77 (75)	
Male	104 (31)	78 (33)	26 (25)	
Disease duration, years	7.0 (2.7, 11.4)	7.4 (3.5, 11.7)	6.3 (2.2, 8.8)	.53
Provider type				.63
NP/PA	230 (68)	160 (68)	70 (68)	
Physician	90 (27)	61 (26)	29 (28)	
Fellow	19 (6)	15 (6)	4 (4)	
Comorbidity				
Fibromyalgia	28 (8)	10 (4)	18 (17)	< .001
Depression	101 (30)	61 (26)	40 (39)	.02
Osteoarthritis	184 (54)	121 (51)	63 (61)	.09
Sleep apnea	63 (19)	47 (20)	16 (16)	.34
Obesity (BMI ≥ 30 kg/m^2^)	130 (42)	87 (39)	43 (47)	.19
BMI, kg/m^2^	28.5 (25.1–33.7)	28.4 (24.2–32.9)	29.4 (26.5–35.4)	.03
Disease assessment				
Patient global, 0-100	33 (11–57)	20 (6–44)	57 (46–72)	< .001
Provider global, 0-100	15 (5–30)	15 (5–40)	15 (10–20)	.20
Pain VAS, 0-100	38 (15–63)	24 (9–50)	60 (44–73)	< .001
Tender joint count ≥ 2	121 (36)	75 (32)	46 (45)	.02
Swollen joint count ≥ 2	122 (36)	82 (35)	40 (39)	.47
DAS28-CRP	3.0 (2.2–4.4)	2.6 (1.9–4.1)	3.7 (2.7–4.7)	.004
CDAI	7.5 (3.0–14.5)	5.6 (2.0–13.8)	9.8 (6.5–15.6)	< .001
Remission, < 2.8	80 (24)	78 (33)	2 (2)	< .001
LDA, ≥ 2.8 to < 10.0	130 (38)	79 (33)	51 (50)	
MDA, ≥ 10.0 to < 22.0	89 (26)	51 (22)	38 (37)	
HDA, ≥ 22.0	40 (12)	28 (12)	12 (12)	
Laboratory assessment				
CRP, mg/L	3.2 (2.9–9.2)	3.3 (2.9–9.4)	3.2 (2.9–9.2)	.96
RF positivity	233 (71)	173 (75)	60 (61)	.01
Anti-CCP antibody positivity	201 (67)	146 (71)	55 (59)	.045
Radiographic joint erosion	172 (52)	133 (57)	39 (39)	.002
Medication use				
Prednisone	150 (44)	101 (43)	49 (48)	.42
Methotrexate	215 (63)	148 (63)	67 (65)	.68
Biologics	125 (37)	83 (35)	42 (41)	.33
Change of RA medications at index visit	91 (27)	69 (29)	22 (21)	.13
Opioid	78 (23)	45 (19)	33 (32)	.009
Fibromyalgia med	33 (10)	16 (7)	17 (17)	.005
Sleep aid	36 (11)	18 (8)	18 (17)	.007
Antidepressant or anxiolytic	80 (24)	45 (19)	35 (34)	.003

^a^Values are presented as median (interquartile range) or number (percentage)

^b^Discordant group contains only those patients with a global assessment greater than the physician global assessment

*anti-CCP* anticyclic citrullinated peptide, *BMI* body mass index, *CDAI* Clinical Disease Activity Index, *CRP* C-reactive protein, *DAS28-CRP* Disease Activity Score in 28 joints using C-reactive protein, *DMARD* disease-modifying antirheumatic drug, *HDA* high disease activity, *LDA* low disease activity, *MDA* moderate disease activity, *NP* nurse practitioner, *PA* physician assistant, *RA* rheumatoid arthritis, *RF* rheumatoid factor

The rheumatology provider at the most recent clinical visit of a patient was a nurse practitioner or physician assistant for 230 patients (68%), attending physician for 90 (27%), and a fellow for 19 (6%), with no significant differences between the discordant and concordant groups (Table 1). The discordant group had a higher median global assessment of disease activity than the concordant group (57 vs. 20; *P* < .001), whereas provider global assessments were similar between the groups. According to the CDAI, a smaller proportion of patients in the discordant group was in remission than in the concordant group (2% vs. 33%), and greater proportions of patients in the discordant group were in LDA (50% vs. 33%) and MDA (37% vs. 22%) categories than in the concordant groups (*P* < .001), while proportions of patients with HDA were similar between groups.

Comparison of patient characteristics between concordant and discordant groups showed no significant differences in age, sex, or provider type (Table 1). The discordant group reported higher pain by VAS than the concordant group (median 60.0 vs. 23.5; *P* < .001). Negative results for RF (*P* = .01) and anti-CCP antibodies (*P* = .045), lack of radiographic joint erosions (*P* = .002), and presence of ≥ 2 tender joints (*P* = .02) were significantly associated with patient-provider discordance. No significant differences were observed between the groups in swollen joint counts or inflammatory markers. Among the comorbidities, fibromyalgia (*P* < .001) and depression (*P* = .02) showed significant association with discordance. However, excluding patients with fibromyalgia did not affect the association of pain VAS with positive discordance (mean pain VAS of 56 in the discordant group vs. 31 in the concordant group; *P* < .001). Current use of opioids (32% vs. 19%, *P* = .009), fibromyalgia medications (17% vs. 7%, *P* = .005), antidepressants or anxiolytics (34% vs. 19%, *P* = .003), and sleep aids (17% vs. 8%, *P* = .007) was significantly higher in the discordant group. Both groups had similar treatment with conventional and biologic DMARDs, without significant differences in DMARD modification at the index visit.

Of 140 patients approached for the prospective substudy, 70 patients agreed to participate, including 50 patients from the discordant group and 20 from the concordant group. Four patients in the discordant group were among the 11 in the study population with lower discordance who were excluded, leaving 46 patients in the discordant group for analysis. In the discordant group, the disease of two patients was in remission, and 26 had LDA, 13 had MDA, and 5 had HDA. The discordant group reported higher median scores for pain VAS (49.0 vs. 25.0, *P* = .002), SF-MPQ-2 continuous pain (3.3 vs. 2.2, *P* = .03), neuropathic pain (2.0 vs. 1.2, *P* = .045), Fibromyalgia Survey WPI (7.0 vs. 4.5, *P* = .008), and polysymptomatic distress (11.5 vs. 7.5, *P* = .007) than the concordant group (Table 2). Of note, fatigue measures were not significantly different between the groups, either by Bristol Rheumatoid Arthritis Fatigue or PROMIS Fatigue Survey. Five patients (10.8%) in the discordant group had PHQ-9 scores ≥ 10, indicating moderate to severe clinical depression. An investigator (JMD) screened these five patients, and one patient consented to psychiatric evaluation on a nonurgent basis; the other patients chose to follow-up with their primary rheumatologist or primary care provider. None expressed suicidal ideation or required urgent evaluation.

**Table 2:** Comparison of patient-reported outcomes between the concordant and discordant groups in the prospective substudy

Patient-reported outcome	Concordant group (*n* = 20)	Discordant group^a^ (*n* = 46)	*P* value
Pain VAS	25.0 (11.0–42.5)	49.0 (28.0–70.0)	.002
SF-MPQ-2 pain score			
Continuous	2.2 (1.8)	3.3 (2.0)	.03
Intermittent	1.8 (2.2)	1.8 (2.0)	.71
Neuropathic	1.2 (1.4)	2.0 (1.8)	.045
Affective	1.1 (1.4)	1.7 (2.0)	.06
Total	1.2 (0.4–2.7)	2.1 (1.2–2.9)	.09
Fibromyalgia Research Survey			
WPI	4.5 (2.5–7.0)	7.0 (5.0–10.0)	.008
SS score	3.5 (2.0–5.5)	4.5 (3.0–6.0)	.05
Polysymptomatic distress	7.5 (5.5–12.5)	11.5 (9.0–16.0)	.007
BRAF score	5.0 (3.8–5.5)	5.3 (4.3–6.7)	.10
PROMIS			
Fatigue	2.4 (1.4–3.4)	2.8 (2.0–3.8)	.22
Pain interference	1.7 (1.3–2.6)	2.2 (1.6–3.1)	.04
Sleep disturbance	2.0 (1.4–3.4)	2.8 (2.0–3.8)	.09
Ability to participate	3.9 (3.4–4.5)	3.5 (3.0–4.1)	.06
HAQ-II disability	0.4 (0.1–1.1)	1.0 (0.5–1.4)	.003
MAAS	4.6 (4.2–5.3)	5.1 (4.5–5.4)	.19
PHQ-9	2.5 (1.0–6.0)	4.0 (3.0–6.0)	.18
GAD-7	1.0 (0.0–1.5)	1.0 (0.0–3.0)	.12

Values are presented as median (interquartile range)

^a^ Discordant group contains only those patients with patient global assessment greater than physician global assessmen

*BRAF* Bristol Rheumatoid Arthritis Fatigue, *GAD-7* Generalized Anxiety Disorder 7, *HAQ-II* Health Assessment Questionnaire II, *MAAS* Mindful Attention Awareness Scale, *PHQ-9* Patient Health Questionnaire 9, *PROMIS* Patient-Reported Outcome Measurement Information System, *SF-MPQ-2* Short-Form McGill Pain Questionnaire 2, *SS* Symptom Severity, *VAS* visual analog scale, *WPI* Widespread Pain Index

To address the possibility of confounding of patient-reported outcome analyses by disease activity states, the next analysis separately compared individual patient-reported outcomes between discordant and concordant groups among patients with LDA versus MHDA (Table 3). Irrespective of disease activity category, the discordant group had higher median scores for pain VAS (for LDA, 37.0 vs. 18.5, *P* = .006; for MHDA, 66.0 vs. 36.0, *P* = .02) and higher median HAQ-II disability scores (for LDA, 0.9 vs. 0.2, *P* = .001; for MHDA, 1.2 vs. 0.9, *P* = .10). In the LDA category, the discordant group had impaired ability to function in activities and social roles according to the PROMIS ability to participate instrument (median, 3.5 vs. 4.3; *P* = .02) and higher PHQ-9 depression scores (median, 4.0 vs. 1.0, *P* = .04) compared with patients in the concordant group. In the MHDA category, patients in the discordant group had higher median scores for fibromyalgia WPI (8.0 vs. 4.0; *P* = .005) and polysymptomatic distress (13.5 vs. 7.5; *P* = .01).

**Table 3:** Comparison of patient-reported outcomes between the groups according to clinical disease activity level

	Remission/low disease activity^a^			Moderate-to-high disease activity^a^		
Measure	Concordant group (*n* = 10)	Discordant group (*n* = 28)	*P* value	Concordant group (*n* = 10)	Discordant group (*n* = 18)	*P* value
Pain VAS	18.5 (3.0–26.0)	37.0 (24.0–56.0)	.006	36.0 (14.0–50.0)	66.0 (56.0–74.0)	.02
HAQ-II	0.2 (0.0–0.3)	0.9 (0.5–1.2)	.001	0.9 (0.4–1.2)	1.2 (0.7–1.2)	.10
SF-MPQ-2	0.7 (0.3–1.6)	1.4 (0.8–2.6)	.10	1.9 (1.0–2.7)	2.3 (1.8–3.9)	.18
WPI	5.0 (1.0–8.0)	6.0 (4.0–9.0)	.18	4.0 (3.0–7.0)	8.0 (6.0–10.0)	.005
SS score	2.5 (1.0–4.0)	4.0 (3.0–6.0)	.11	4.0 (2.0–6.0)	6.0 (3.0–8.0)	.16
PSD	8.0 (2.0–12.0)	9.0 (8.0–15.0)	.13	7.5 (6.0–13.0)	13.5 (10.0–16.0)	.01
BRAF score	4.5 (3.7–5.3)	5.2 (4.3–6.3)	.23	5.0 (4.0–6.0)	6.3 (4.3–7.0)	.28
PROMIS						
Pain interference	1.3 (1.1–1.8)	1.9 (1.5–2.4)	.05	2.2 (1.6–2.8)	2.9 (2.1–3.5)	.05
Ability to participate	4.3 (3.8–5.0)	3.5 (3.1–4.2)	.02	3.7 (3.1–4.0)	3.3 (2.6–4.0)	.43
Sleep disturbance	1.6 (1.3–2.3)	2.1 (1.8–2.9)	.06	2.3 (1.9–2.8)	2.5 (1.8–3.3)	.50
Fatigue	1.8 (1.0–3.4)	2.6 (2.0–3.5)	.10	2.9 (2.3–3.4)	3.1 (2.3–3.8)	.43
MAAS	5.0 (4.5–5.6)	5.1 (4.6–5.4)	.95	4.4 (4.1–4.9)	5.3 (4.5–5.5)	.07
PHQ-9	1.0 (0.0–5.0)	4.0 (2.0–6.0)	.04	4.0 (2.0–11.0)	4.0 (3.0–6.0)	>.99
GAD-7	0.0 (0.0–1.0)	1.0 (0.0–3.0)	.06	1.5 (0.0–4.0)	1.5 (1.0–6.0)	.71

Values are presented as median (interquartile range)

^a^Disease activity level was classified according to the Clinical Disease Activity Index

*BRAF* Bristol Rheumatoid Arthritis Fatigue, *FRS* Fibromyalgia Research Survey, *GAD-7* Generalized Anxiety Disorder 7, *HAQ-II* Health Assessment Questionnaire II, *MAAS* Mindful Attention Awareness Scale, *PHQ-9* Patient Health Questionnaire 9, *PROMIS* Patient-Reported Outcomes Measurement Information System, *PSD* polysymptomatic distress, *SF-MPQ-2* Short-Form McGill Pain Questionnaire 2, *SS* Symptom Severity, *VAS* visual analog score, *WPI* Widespread Pain Index

In order to explore the different effects of depression and fibromyalgia on discordance depending on disease activity states, the analysis was also performed on the overall study population (Table 4). In the LDA category, a higher prevalence of depression in this analysis by provider diagnosis was evident in the discordant group (47% vs. 27%, *P* = .006), but in the MHDA category there was no difference in the prevalence of depression between the discordant and concordant groups (30% vs. 24%, *P* = .46). As compared to the abovementioned observations for the fibromyalgia WPI, a higher prevalence of fibromyalgia by provider diagnosis was observed in the discordant groups in both the LDA (15% vs. 3%, *P* = .002) and the MHDA (20% vs. 6%, *P* = .015) categories.

**Table 4:** Comparison of patient characteristics between the discordant and concordant groups according to clinical disease activity level in the overall retrospective study population^a^

	Remission/low disease activity			Moderate-to-high disease activity		
Characteristic	Concordant group (*n* = 157)	Discordant group (*n* = 53)	*P* value	Concordant group (*n* = 79)	Discordant group (*n* = 50)	*P* value
Age, years	64.9 (55.1–74.3)	66.3 (59.8–72.3)	.46	61.1 (55.1–71.5)	59.0 (50.3–71.0)	.23
Sex			.70			.15
Female	102 (65%)	36 (68%)		56 (71%)	41 (82%)	
Male	55 (35%)	17 (32%)		23 (29%)	9 (18%)	
Disease duration, years	7.0 (5.2–11.8)	7.9 (3.1–11.1)	.84	8.8 (2.2–11.6)	5.2 (1.6–7.8)	.47
Provider type			.60			.90
NP/PA	108 (69%)	39 (74%)		52 (66%)	31 (62%)	
Physician	37 (24%)	12 (23%)		24 (30%)	17 (34%)	
Fellow	12 (8%)	2 (4%)		3 (4%)	2 (4%)	
Comorbidities						
Fibromyalgia	5 (3%)	8 (15%)	.002	5 (6%)	10 (20%)	.018
Depression	42 (27%)	25 (47%)	.006	19 (24%)	15 (30%)	.46
Osteoarthritis	76 (48%)	39 (74%)	.001	45 (57%)	24 (48%)	.32
Sleep apnea	26 (17%)	10 (19%)	.70	21 (27%)	6 (12%)	.047
Obesity (BMI ≥ 30 kg/m^2^)	50 (34%)	23 (47%)	.12	37 (48%)	20 (48%)	.96
BMI (kg/m^2^)	27.6 (23.7–31.8)	29.0 (26.8–34.0)	.017	29.8 (25.8–35.4)	29.6 (26.1–38.3)	.75
Disease assessments						
Patient global (0–100)	10 (4–22)	50 (40–60)	<.001	49 (27–68)	66 (55–77)	< .001
Provider global (0–100)	10 (5–15)	10 (5–10)	.97	45 (30–60)	20 (15–30)	< .001
Pain (0–100 mm)	15 (5–28)	50 (37–66)	<.001	55 (32–76)	66 (57–80)	.007
Tender joint count ≥ 2	12 (8%)	9 (17%)	.05	63 (80%)	37 (74%)	.45
Swollen joint count ≥ 2	21 (31%)	17 (13%)	.98	61 (77%)	33 (66%)	.16
DAS28-CRP	1.9 (1.7–6.0)	2.6 (2.5–2.7)	.002	4.1 (3.6–5.0)	4.4 (3.4–5.0)	.98
CDAI	2.9 (1.1–5.5)	6.8 (5.5–8.7)	<.001	18.9 (13.6–30.8)	15.7 (12.0–21.5)	.026
Laboratory assessments						
CRP, mg/L	3.0 (2.9–7.7)	2.9 (2.9–6.6)	.58	4.1 (2.9–14.7)	5.4 (2.9–9.5)	.97
RF, positive	115 (76%)	29 (58%)	.017	58 (74%)	31 (65%)	.24
ACPA, positive	93 (69%)	27 (56%)	.10	53 (74%)	28 (62%)	.19
Radiographic joint erosions	96 (62%)	18 (35%)	.001	37 (47%)	21 (44%)	.69
Medication use						
Prednisone	62 (39%)	21 (40%)	.99	39 (49%)	28 (56%)	.46
Methotrexate	94 (60%)	40 (75%)	.041	54 (68%)	27 (54%)	.10
Biologics	52 (33%)	18 (34%)	.91	31 (39%)	24 (48%)	.33
Change of RA medications at index visit?	34 (22%)	6 (11%)	.10	35 (44%)	16 (32%)	.16
Opioid	10 (6%)	5 (9%)	.16	12 (15%)	11 (22%)	.32
Fibromyalgia medication	8 (5%)	6 (11%)	.12	8 (10%)	11 (22%)	.06
Sleep aid	12 (8%)	9 (17%)	.05	6 (8%)	9 (18%)	.07
Anti-depressant or anxiolytic	28 (18%)	21 (40%)	.001	17 (22%)	14 (28%)	.40

Values are median (interquartile range) or number (%)

^a^Discordant group contains only those with patient global assessment greater than physician global assessment

*ACPA* anti-cyclic citrullinated peptide antibody, *BMI* body mass index, *CDAI* Clinical Disease Activity Index, *CRP* C-reactive protein, *DAS28-CRP* Disease Activity Score in 28 joints using C-reactive protein, *NP* nurse practitioner, *PA* physician assistant, *RA* rheumatoid arthritis, *RF* rheumatoid factor

Multivariable logistic regression analysis in the total retrospective population showed that diagnoses of fibromyalgia (adjusted odds ratio (OR) 3.06, 95% confidence interval (CI) 1.87–8.00), depression (adjusted OR 1.79, 95% CI 1.02–3.15), and lack of erosions (adjusted OR 0.56, 95% CI 0.32–0.97) were independently associated with patient-provider discordance (Table 5). The associations of body mass index and osteoarthritis with discordance did not reach statistical significance in the overall population. However, osteoarthritis was significantly associated with discordance in the LDA category (adjusted OR 3.36, 95% CI 1.35–8.34) but not in the MDHA category (adjusted OR 0.86, 95% CI 0.33–2.27). The addition of use of glucocorticoids, conventional or biologic DMARDs, opioids, fibromyalgia medications, and antidepressants or anxiolytics to this model did not reveal any significant associations. Pain VAS was not added to the final model for the purposes of this study due to colinearity with the patient global assessment as well as other variables in the model (e.g., fibromyalgia). Overall, the model showed strong performance, with a *C* statistic of 0.694.

**Table 5:** Multivariable logistic regression model of patient-physician discordance compared with concordance in assessments of global disease activity

	Odds ratio (95% CI)		
Characteristic	Overall	Remission/low disease activity	Moderate-to-high disease activity
Fibromyalgia	3.06 (1.17–8.00)	4.26 (0.92–19.59)	2.56 (0.67–9.73)
Osteoarthritis	1.81 (0.98–3.36)	3.36 (1.35–8.34)	0.86 (0.33–2.27)
Depression	1.79 (1.02–3.15)	3.16 (1.43–6.98)	1.10 (0.43–2.82)
BMI, kg/m^2^	1.03 (0.99–1.07)	1.07 (1.00–1.14)	1.00 (0.95–1.06)
Age, per year	1.00 (0.97–1.02)	1.00 (0.97–1.04)	0.99 (0.96–1.02)
Male sex	0.79 (0.43–1.45)	1.29 (0.56–2.98)	0.60 (0.22–1.63)
RF or anti-CCP antibody positivity	0.67 (0.35–1.28)	0.76 (0.30–1.89)	0.50 (0.18–1.36)
Radiographic joint erosion present	0.56 (0.32–0.97)	0.34 (0.16–0.74)	1.17 (0.50–2.76)

*CI* confidence interval, *anti-CCP* anticyclic citrullinated peptide, *BMI* body mass index, *RF* rheumatoid factor

**Fig. 2:**
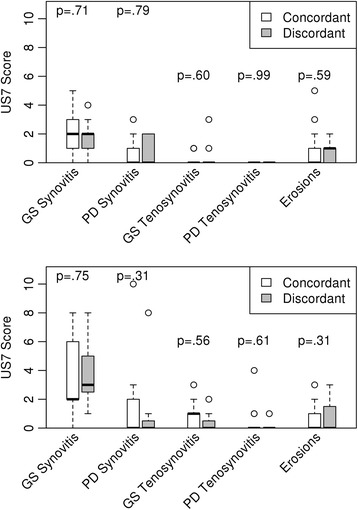
Comparison of ultrasonographic assessments of disease activity according to the Ultrasound 7 (*US7*) scoring method between concordant and discordant groups with rheumatoid arthritis. US7 scores of gray scale (*GS*) and power Doppler (*PD*) synovitis, GS and PD tenosynovitis, and erosions for concordant and discordant groups, stratified by LDA (*upper panel*) vs MHDA (*lower panel*). Error bars indicate standard deviations

Comparison of Ultrasound 7 scores between concordant and discordant groups was stratified by disease activity categories (Fig. 2). The ultrasound studies were performed for research purposes as part of the prospective substudy and were not available to the primary rheumatologist who completed the provider global assessment, so the Ultrasound 7 scores were independent of the determination of patient-provider discordance. No statistically significant differences were found in continuous scores for GS synovitis, PD synovitis, GS tenosynovitis, PD tenosynovitis, or GS erosions between the groups in the LDA or MHDA categories (*P* > .10 for all comparisons). Among patients with LDA, active GS synovitis (≥ 2) was detected in 60% of the concordant and discordant groups (*P* = .97), and active PD synovitis (≥ 2) was detected in 30% of the concordant group and 14% of the discordant group (*P* = .27). Among patients with MHDA in the concordant group vs. discordant group, active GS synovitis (≥ 2) was detected in 90% and 78% (*P* = .42) and active PD synovitis (≥) was detected in 10% and 33% (*P* = .17).

To evaluate the potential for participation bias, the 70 participants in the prospective substudy were compared with 70 patients who declined to participate (Table 6). No significant differences were seen between participants and nonparticipants in discordance frequency, demographic characteristics, highest education level, provider type, or comorbidities. Participants were less likely than nonparticipants to have positive RF (53% vs. 76%, *P* = .004), anti-CCP antibodies (53% vs. 73%, *P* = .02), elevated CRP level (19% vs. 36%, *P* = .04), and radiographic joint erosions (35% vs. 54%, *P* = .03). However, no significant differences were found in pain, HAQ-II disability, clinical disease activity measures, or treatments between participants and nonparticipants.

**Table 6:** Comparison of clinical characteristics between participants and patients who declined participation in the prospective substudy

Characteristic	Participants (*n* = 70)	Nonparticipants (*n* = 70)	*P* value
Study group			.70
Discordant	50 (71)	51 (73)	
Concordant, MHDA	10 (14)	7 (10)	
Concordant, LDA	10 (14)	12 (17)	
Age, years	63 (51–71)	59 (53–71)	.52
Sex			.56
Female	51 (73)	54 (77)	
Male	19 (27)	16 (23)	
Highest level of schooling completed			.46
Information missing	5	6	
Grade 8 or less	0 (0)	1 (2)	
Some high school but did not graduate	4 (6)	3 (5)	
High school graduation or GED	15 (23)	23 (36)	
Some college or 2-year degree	25 (38)	21 (33)	
4-year college degree	10 (15)	10 (16)	
Postgraduate studies	11 (17)	6 (9)	
Residence			.32
Minnesota	70 (100)	69 (99)	
Iowa	0 (0)	1 (1)	
Rochester, Minnesota	29 (41)	28 (41)	.92
Provider type			.38
Attending physician	20 (29)	18 (26)	
Fellow	1 (1)	4 (6)	
NP/PA	49 (70)	48 (69)	
Comorbidity			
BMI (kg/m^2^)	29.7 (19.5–47.7)	28.9 (26.2–34.1)	.91
Degenerative joint disease	41 (59)	35 (50)	.31
Fibromyalgia	12 (17)	5 (7)	.07
Obstructive sleep apnea	9 (13)	10 (14)	.81
Depression	23 (33)	21 (30)	.72
Anxiety	6 (9)	7 (10)	.77
Disease activity assessment			
Tender joint count	0.5 (0.0–4.0)	1.0 (0.0–5.0)	.98
Swollen joint count	0 (0.0–4.0)	1.0 (0.0–4.0)	.53
HAQ-II disability index, 0–3	0.7 (0.3–1.1)	0.7 (0.0–1.3)	.75
Pain, 100 mm VAS	54 (23–68)	52 (25–70)	.87
Patient global assessment, 0–100	49 (29–60)	50 (30–70)	.25
Provider global assessment, 0–100	15 (0–80)	15 (0–100)	.71
DAS28-CRP	2.8 (2.4–3.5)	3.6 (2.5–4.6)	.33
CDAI	9.8 (5.7–17.7)	11.1 (6.0–17.4)	.69
CDAI Categories			.47
Remission	10 (14)	6 (9)	
LDA	28 (40)	26 (37)	
MDA	21 (30)	29 (41)	
HDA	11 (16)	9 (13)	
Laboratory testing			
Rheumatoid factor positivity	36 (53)	52 (76)	**.004**
Anti-CCP positivity	33 (53)	47 (73)	**.02**
ANA positivity	18 (31)	13 (23)	.35
CRP at index visit, mg/L	2.9 (1.6–46.1)	5.3 (2.9–11.0)	**.003**
Abnormal CRP concentration (≥ 8 mg/L)	11 (19)	20 (36)	**.04**
ESR at index visit, mm/h	9 (3–17)	10 (3,–21)	.50
Abnormal ESR (i.e., > 22 male and > 29 female)	6 (11)	11 (20)	.22
Radiographic erosion present	24 (35)	37 (54)	**.03**
Medication			
Prednisone	25 (36)	39 (56)	**.02**
Methotrexate	51 (73)	41 (59)	.08
Nonmethotrexate DMARD	27 (39)	25 (36)	.73
TNF inhibitor	18 (26)	19 (27)	.85
Any biologic	21 (30)	31 (44)	.08
Opioid	16 (23)	18 (26)	.31
Tramadol	7 (10)	12 (17)	.22
Gabapentin	9 (13)	4 (6)	.15
Pregabalin	1 (1)	0 (0)	.31
Duloxetine	2 (3)	2 (3)	> .99
Fibromyalgia medication	12 (17)	6 (9)	.13
NSAID	26 (37)	35 (50)	.13
Sleep aid	11 (16)	7 (10)	.31
Antidepressant or anxiolytic	18 (26)	22 (31)	.45
DMARD modification at index visit?			.10
No	59 (84)	51 (73)	
Yes	11 (16)	19 (27)	

Values are presented as median (interquartile range) or number (percentage) of patients

Significant results are indicated in bold typeface

*ANA* antinuclear antibodies, *anti-CCP* anticyclic citrullinated peptide, *BMI* body mass index, *CDAI* Clinical Disease Activity Index, *CRP* C-reactive protein, *DAS28-CRP* Disease Activity Score in 28 joints using C-reactive protein, *DMARD* disease-modifying antirheumatic drug, *ESR* erythrocyte sedimentation rate, *GED* general education development, *HAQ-II* Health Assessment Questionnaire II, *HDA* high disease activity, *LDA* low disease activity, *MDA* moderate disease activity, *MHDA* moderate-to-high disease activity, *NP* nurse practitioner, *NSAID* nonsteroidal anti-inflammatory drug, *PA* physician assistant, *TNF* tumor necrosis factor, *VAS* visual analog scale

## Discussion

This study is among the first to perform a comprehensive clinical evaluation of the myriad potential correlates of patient-provider discordance—including several domains not previously assessed—in a real-world RA population. Overall, the prevalence of patient-provider discordance in this study was slightly less, at 33%, than the pooled estimate of 43% reported in a recent systematic review and meta-analysis [1]. The prevalence of discordance in which patients rate their disease as more severe than their providers (i.e., positive discordance) was 29%, which is nearly identical to studies by Barton et al. [3] and Khan et al. [2] at 29% and 30% of clinical encounters. In particular, fibromyalgia, depression, and nonerosive disease were independently associated with patient-provider discordance.

Barton et al. [3] have previously shown that positive discordance affects categorization of disease activity by composite measures, such that removal of the patient global assessment and calculation of the three-variable DAS28 led to shifting of patients from MHDA to LDA categories. The present study demonstrates that patients with positive discordance are less likely to be in remission and more likely to be in the LDA or MDA categories. Together, the findings underscore the difficulty in interpreting composite disease activity scores in the clinical setting of patient-provider discordance, considering the absence of meaningful correlation between discordance and inflammatory measures, as well as the uncertainties with implementation of current treat-to-target recommendations [23, 24].

Pain intensity is a key correlate of patient-provider discordance [1–3, 7, 25], but few studies have addressed specific characteristics or comorbidities related to pain etiologies. Based on the results of the SF-MPQ-2 analyses, continuous and neuropathic pain types are associated with patient-provider discordance in RA. The findings of this study are in agreement with Koop et al. [26], who reported neuropathic pain characteristics in patients with RA using the pain DETECT questionnaire.

Fibromyalgia prevalence ranges from 12% to 20% among RA patients, with an estimated incidence of 5 per 100 patient-years [27–30]. Ranzolin and colleagues [31] have shown that patients with RA and concomitant fibromyalgia have higher pain scores than RA patients without fibromyalgia, yet they have relatively low provider global assessments. Their study did not report patient global assessments. Khan et al. [2] reported in the Quantitative Standard Monitoring of Patients With Rheumatoid Arthritis study that 4.6% of the positive discordance group had investigator-reported fibromyalgia compared with 2.5% in the concordant group, which was statistically significant. The present finding of a 17% prevalence of fibromyalgia diagnosis by the treating physician in the discordant group is considerably higher than in the study by Khan et al. but certainly is consistent with the overall prevalence of fibromyalgia in RA, highlighting the clinical significance of previous data on fibromyalgia to patient-provider discordance. Previous studies have demonstrated that, among RA patients, fibromyalgia is associated with higher DAS28 and adverse scores for functional ability and health-related quality of life (HRQOL) [3, 30, 32, 33]. Data also show that patients with positive discordance may be overtreated with biologic therapies to which they are unlikely to respond [32]. Considering current concepts of centralized pain in patients with fibromyalgia, the data suggest that abnormal central pain processing may be the key driver of chronic widespread pain among patients with RA in the setting of positive patient-provider discordance and may also explain some inadequate responses to DMARD therapy [26, 34, 35].

Depression is also prevalent in RA patients and has been studied extensively in this population [36]. Barton et al. [3] showed that depression is strongly associated with patient-provider discordance. In their study, the frequency of depression as defined by a PHQ-9 score ≥ 10 among the population with positive discordance was 43%, which is similar to the frequency of clinical depression of 39% in the present study. Results of the multivariable analysis suggest that pain, fibromyalgia, and depression make complementary contributions to patient-provider discordance. Osteoarthritis and elevated body mass index appear also to make a small contribution to positive discordance, mainly in LDA states.

Indeed, comparison of patient-reported outcomes between the discordant and concordant groups separately in remission or with LDA versus MHDA suggests that depression and fibromyalgia have distinct roles in mediating patient-provider discordance. In LDA states, pain intensity and pain-related interference in activities and role functions, fibromyalgia, and depression are complementary mediators of positive discordance. In MHDA states, fibromyalgia as defined by the WPI and polysymptomatic distress are key determinants of discordance whereas depression has no effect. Interpretation of these findings must consider the differences in the definitions of fibromyalgia between the analyses shown in Table 3 (fibromyalgia WPI) and Table 4 (previous diagnosis of fibromyalgia). The findings suggest that the activity or severity of fibromyalgia is important, meaning that milder or partially treated fibromyalgia may be mediating discordance in LDA states and more active or severe fibromyalgia may be driving discordance in MHDA states. Wolfe [37] coined the term *fibromyalgianess*, noting that the distribution of polysymptomatic distress does not suggest a discrete entity but rather a continuous spectrum of illness. Perhaps the findings of the present study indicate interactions between higher inflammatory activity and abnormal pain processing in the development of complex, disease-related centralized pain and polysymptomatic distress [38, 39].

As suggested by Wolfe et al. [39], consideration should be given to disaggregation of domains within the patient global assessment to develop management pathways targeting optimal patient-centered outcomes. For example, high PHQ-9 scores in the present study identified several patients with undiagnosed depression. The findings suggest that routine measurement of patient-reported outcomes could help identify the central drivers of adverse health status apart from inflammatory disease activity and thereby could suggest potential interventions. Further research is necessary to develop a feasible, time-efficient set of patient-reported outcomes and determine how to integrate them into typical practice settings. In the meantime, rheumatologists may consider implementation of the tools reported in this study. The patient-reported outcome measures used in this study may be obtained at the following websites: for PROMIS, http://www.healthmeasures.net/explore-measurement-systems/promis; PHQ-9, http://www.phqscreeners.com/select-screener; and for the fibromyalgia WPI and SS score, https://www.rheumatology.org/Practice-Quality/Clinical-Support/Criteria/ACR-Endorsed-Criteria.

Ultrasonography is a more sensitive measure of disease activity than clinical examination [40]. In the present study, although patients in the discordant group commonly had active synovitis, ultrasonography-defined synovitis did not discriminate the groups. Unrecognized disease activity does not appear to be a major factor in discordance. Nonetheless, this tool could be useful in evaluating disease activity and guiding disease-modifying therapy in patients with patient-provider discordance, considering that composite disease activity scores are less reliable in this clinical setting [41].

### Limitations

Previous studies have emphasized differences between the patient global assessment of disease activity and the patient general health assessment [4, 42]. The question for the patient global assessment in the present study did not specifically ask about joint tenderness, swelling, or inflammation, but the wording was similar to previous studies [3, 43]. The cross-sectional design prevented assessment of the persistence of discordance over time, as well as causal associations. Future studies are necessary to understand the clinical factors leading to the development of discordance. Morning stiffness is an important symptom of RA but was not assessed in this study. The results show some evidence of selection bias in the prospective substudy, in which patients who chose to participate were somewhat less likely to have positivity for RF and anti-CCP antibodies and erosive disease than nonparticipants. However, participants and nonparticipants were otherwise similar for pain, disability, and clinical disease activity, so it is unlikely that this minor selection bias had a major impact on the patient-reported outcome and ultrasonography results. Speculatively, patients with seronegative RA may perceive greater uncertainty on the part of their providers, leading them to be more interested in participating in a study on patient-provider discordance. Discordance was not fully explained by the model used in this study, and in view of previous findings [43] it is an important limitation that patient education, health literacy, and patient-physician communication were not assessed in this study. Finally, several factors may limit the generalizability of the findings of this study, such as the site at an academic referral center with substantial clinical subspecialization, as well as the racial and ethnic homogeneity of the study population.

## Conclusions

The contribution of this study is a comprehensive, patient-level description of the clinical phenotypes that are associated with patient-provider discordance. This study should inform the selection and testing of patient-reported outcomes for routine evaluation of discordance. The findings should inform the development of a standardized approach to evaluation and management, as well as enhancement of patient-provider communication and shared decision making for RA patients in the scenario of discordance. At this time, it would be prudent for rheumatology care providers to diagnose and treat comorbidities, such as depression and fibromyalgia, using available pharmacologic and nonpharmacologic therapies and to monitor the impact on the health status of patients.

## Abbreviations

ACR/EULAR: American College of Rheumatology/European League Against Rheumatism
anti-CCP: Anticyclic citrullinated peptide
CDAI: Clinical Disease Activity Index
CI: Confidence interval
DAS28: Disease Activity Score 28
DAS28-CRP: Disease Activity Score 28 using C-reactive protein
DMARD: Disease-modifying antirheumatic drug
GS: Gray scale
HAQ-II: Health Assessment Questionnaire-II
HDA: High disease activity
HRQOL: Health-related quality of life
LDA: Low disease activity
MDA: Moderate disease activity
MHDA: Moderate-to-high disease activity
OR: Odds ratio
PD: Power Doppler
PHQ-9: Patient Health Questionnaire 9
PROMIS: Patient-Reported Outcomes Measurement Information System
RA: Rheumatoid arthritis
RF: Rheumatoid factor
SF-MPQ-2: Short-Form McGill Pain Questionnaire 2
SS: Symptom Severity
VAS: Visual analog scale
WPI: Widespread Pain Index
